# First complete genome sequence of European turkey coronavirus suggests complex recombination history related with US turkey and guinea fowl coronaviruses

**DOI:** 10.1099/jgv.0.000338

**Published:** 2016-01

**Authors:** P. A. Brown, F. Touzain, F. X. Briand, A. M. Gouilh, C. Courtillon, C. Allée, E. Lemaitre, C. De Boisséson, Y. Blanchard, N. Eterradossi

**Affiliations:** ^1^​ VIPAC Unit, Agence Nationale de Sécurité Sanitaire (ANSES), Laboratoire de Ploufragan-Plouzané, Université Européenne de Bretagne, BP 53-22440 Ploufragan, France; ^2^​ EPICOREM Consortium, Université de Caen, Unité de Recherche Risques Microbiens (U2RM), F-14000 Caen, France; ^3^​ VB Unit, Agence Nationale de Sécurité Sanitaire (Anses), Laboratoire de Ploufragan-Plouzané, Université Européenne de Bretagne, G, BP 53-22440 Ploufragan, France; ^4^​ Institut Pasteur, Environment and Infectious Risks Research and Expertise Unit, 25–28 rue du Docteur Roux, F-75724 Paris Cedex 15, France; ^5^​ Université de Caen, Unité de Recherche Risques Microbiens (U2RM), F-14000 Caen, France

## Abstract

A full-length genome sequence of 27 739 nt was determined for the only known European turkey coronavirus (TCoV) isolate. In general, the order, number and size of ORFs were consistent with other gammacoronaviruses. Three points of recombination were predicted, one towards the end of 1a, a second in 1b just upstream of S and a third in 3b. Phylogenetic analysis of the four regions defined by these three points supported the previous notion that European and American viruses do indeed have different evolutionary pathways. Very close relationships were revealed between the European TCoV and the European guinea fowl coronavirus in all regions except one, and both were shown to be closely related to the European infectious bronchitis virus (IBV) Italy 2005. None of these regions of sequence grouped European and American TCoVs. The region of sequence containing the S gene was unique in grouping all turkey and guinea fowl coronaviruses together, separating them from IBVs. Interestingly the French guinea fowl virus was more closely related to the North American viruses. These data demonstrate that European turkey and guinea fowl coronaviruses share a common genetic backbone (most likely an ancestor of IBV Italy 2005) and suggest that this recombined in two separate events with different, yet related, unknown avian coronaviruses, acquiring their S-3a genes. The data also showed that the North American viruses do not share a common backbone with European turkey and guinea fowl viruses; however, they do share similar S-3a genes with guinea fowl virus.

## Introduction

Coronaviruses, family *Coronaviridae* in the order *Nidovirales*, are the largest known RNA viruses, having a genome length of approximately 30 kb. They are enveloped viruses that form spherical structures between 50 and 200 nm in diameter, with spiked bulbous projections of their S membrane glycoprotein mediating fusion with target cell membranes. They have a capped and polyadenylated positive-sense genome that serves as a template for the synthesis of either a full-length antigenome or a nested set of shorter negative-stranded RNA copies. These, in turn, generate new full-length positive-sense genomic molecules or a nested set of subgenomic mRNAs for expression of the viral proteins (Lai *et al.*, 2007), respectively.

Currently, four genera of coronaviruses have been defined, alpha, beta, gamma and delta. The genus *Gammacoronavirus* is mostly composed of viruses isolated from birds: Galliformes (chicken, turkey, quail, guinea fowl, pheasant, peafowl), Anseriformes (duck, goose teal, swan, pintail), Columbiformes (pigeon), Pelecaniformes (spoonbill, heron), Suliformes (cormorant), Charadriiformes (red knot, oystercatcher, black-headed gull) and Passeriformes (bulbul); however, recently, gammacoronaviruses have also been identified in the beluga whale and bottlenose dolphins (Mihindukulasuriya *et al.*, 2008; Woo *et al.*, 2014). The most economically important of the avian coronaviruses (AvCoVs) are infectious bronchitis virus (IBV) and turkey coronaviruses (TCoVs). IBVs, which were the first coronaviruses to be isolated back in the 1930s, cause highly contagious infectious bronchitis in domestic fowl, a respiratory, renal and genital disease with serious economic consequences worldwide (Cavanagh & Gelb, 2008). TCoVs, initially linked in the 1970s with an enteric disease known as transmissible enteritis, coronaviral enteritis of turkeys or bluecomb (Guy, 2008), have more recently been associated with a syndrome that includes several intestinal disorders occurring in turkeys before 7 weeks of age and usually within the first 3 weeks of life (Barnes *et al.*, 2000), known as poult enteritis complex (PEC). The emergence of coronaviruses in turkeys in the USA was proposed to have resulted from recombination events involving IBVs and an as-yet-unidentified coronavirus donating a spike (S) gene that encoded a protein of low amino acid identity to those of IBV (35 %). This is suspected to have resulted in a host shift from chickens to turkeys and also in an altered tissue tropism of the virus from upper respiratory to intestinal. Recombination has been proposed in preference to evolution or selection of a subpopulation of viruses owing to this low S protein amino acid identity and because under experimental conditions exposure/passage of TCoVs in chickens did not result in selection for genetic changes that were sufficient to maintain infection and replication in chickens (Jackwood *et al.*, 2010). It has also been shown, using patterns of synonymous substitutions to determine the index of mutation rates in protein-coding genes, that mutation rates in the S genes of IBV and TCoVs are very similar to those in their other genes (Hughes, 2011).

Since 2003 there have been an increasing number of turkey flocks in different geographical locations in France exhibiting clinical signs compatible with PEC. Studies performed in France between 1985 and 1989 (Andral & Toquin, 1984a, b) identified mixed infections of reovivuses, rotaviruses, adenoviruses and picorna-like viruses in such cases. Later, coronaviruses were also implicated with the disease in the USA in the 1990s, the UK in 2001, Italy in 2002, and then Brazil in 2007. A study performed in France on samples collected between 2007 and 2009 in 52 flocks with signs of PEC showed coronaviruses to be present in 37 % of these flocks; however, no TCoV was detected in 23 intestinal samples collected in French turkey flocks with enteritis before 1988 (Maurel *et al.*, 2010).

In France in 2008, a coronavirus (Fr TCoV 080385d) was isolated from turkeys exhibiting clinical signs compatible with PEC (Maurel *et al.*, 2009) and its S gene was later shown to form a sublineage related to, but significantly different from, coronaviruses isolated from turkeys in North America (US TCoVs) (Maurel *et al.*, 2011). These conclusions were drawn according to the nucleotide and putative amino composition of its full-length S ORF; 98 % identity at both the nucleotide and amino acid levels was observed between the detected genomes of three French strains, whereas these figures were at most 65 and 60 % with US TCoVs, and at most 50 and 37 % with IBVs. Even lower aa identities were seen in the S1 subdomain between the Fr TCoVs and the US TCoVs (42 %), and between Fr TCoVs and IBV (18 %). Further analysis using partial nucleotide sequences of the nucleocapsid (N) and RNA-dependent RNA polymerase ORFs suggested that similar recombination events played a role in the evolution of the US and Fr TCoVs (Maurel *et al.*, 2011), yet involving IBVs originating from their respective continents.

The full genome sequence for Fr TCoV 080385d has been completed in this study using next-generation sequencing (NGS) (Miseq), allowing complete phylogenetic analysis and thorough assessment of potential recombination events involved in the emergence of Fr TCoVs.

## Results

### Genome organization and sequence overview

**Fig. 1.**
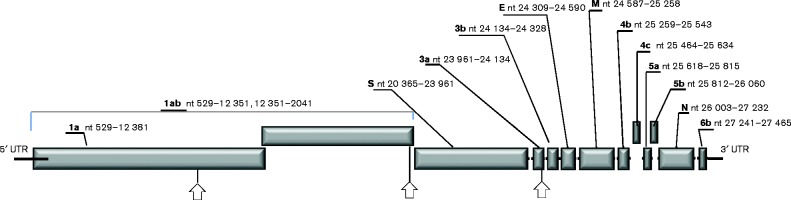
Fr TCoV genome organization (not drawn to scale). Above the graphic the names (bold and underlined) of each ORF are shown, followed by their nucleotide positions (start–stop codons included). Arrows below the graphic correspond to the predicted positions of the most likely recombination sites (positions 8658, 20 280 and 24 058, from left to right).

The full-length sequence was determined using NGS sequencing, supported by endpoint reverse transcription PCR (RT-PCR), 3′ RACE and Sanger sequencing. These sequences where assembled into one continuous sequence of 27 739 nt. In general, the order, number and size of ORFs were consistent with other gammacoronaviruses: following the 5′ untranslated region, which incorporated the IBV and TCoV consensus leader transcriptional regulatory sequence (TRS-L) of CUUAACAA (Bentley *et al.*, 2013), were two large ORFs that overlapped to encode polyproteins 1a (pp1a) and 1ab (pp1ab), then ORFs S, 3a, 3b, E, M, 4b, 4c, 5a, 5b, N and 6b (Fig. 1). Three papain-like and 11 3C-like protease cleavage sites were predicted in pp1ab, which were consistent with those previously described for a number of alpha-, beta- and gammacoronaviruses (Gao *et al.*, 2003). A TRS (as predicted from the genome sequence) CUUAACAA was found upstream of ORFs 1a, M, 5a and N, whereas TRS CUGAACAA was found upstream of ORFs S and 3a, and TRSs UUCAACAA, GUGACCAA and AGGAACAA were found upstream of ORFs E, 4a and 6, respectively. All TRSs of Fr TCoV were identical to those of US TCoV-ATCC and another French coronavirus, pathogenic for guinea fowl (GfCoV/Fr/2011), with the exception of the TRS for 6b (Table 1).

**Table 1.** Comparison of putative TRSs based on genomic sequence data in TCoV and GfCoV/Fr/2011 Accession numbers of viruses studied are shown in Table S1.

Virus	ORF 1a/b	S	3a/b	E	M	4b/c	5a/b	N	6b
Fr-TCoV	CUUAACAA	CUGAACAA	CUGAACAA	UUCAACAA	CUUAACAA	GUGACCAA	CUUAACAA	CUUAACAA	AGGAACAA
TCoV-MG10	––––––––	––––––––	UAU–––––	––––G–––	––––––––	––––––––	–G––––––	––––––––	––––––––
TCoV-VA-74	––––––––	––––––––	UAU–––––	––––G–––	––––––––	––––––––	––––––––	––A–––––	––––––––
TCoV-TX-98	––––––––	––––––––	UAU–––––	––––G–––	––––––––	––––––––	––––––––	––––––––	––––––––
TCoV-TX-GL	––––––––	––––––––	UAU–––––	––––A–––	––––––––	––––––––	–––––A––	––––––––	––––––––
TCoV-IN	––––––––	––––––––	UAU–––––	––––––––	––––––––	––––––––	––––––––	––––––––	––––––––
TCoV-540	––––––––	––––––––	A–––––––	––––––––	––––––––	–U––U–––	––––––––	––––––––	––––––––
TCoV-ATCC	––––––––	––––––––	––––––––	––––––––	––––––––	––––––––	––––––––	––––––––	––––––––
Gf-TCoV		––––––––	––––––––	––––––––	y–––––––	––––––––	––––––––	––––––––	––––C–––

### Recombination events

Sixty-nine full genomes of avian gammacoronaviruses analysed by recombination detection software package RDP4 v4.39 (Martin *et al.*, 2010) predicted three putative recombination breakpoints in the Fr TCoV genome using six different methods with *E*-values ranging between 1.15 × 10^− 24^ and 1.15 × 10^− 180^. One breakpoint was predicted within ORF 1a, a second just upstream of the S ORF and a third within ORF 3a (Fig. 1, arrows).

### Phylogenetic analysis of Fr TCoV

**Fig. 2.**
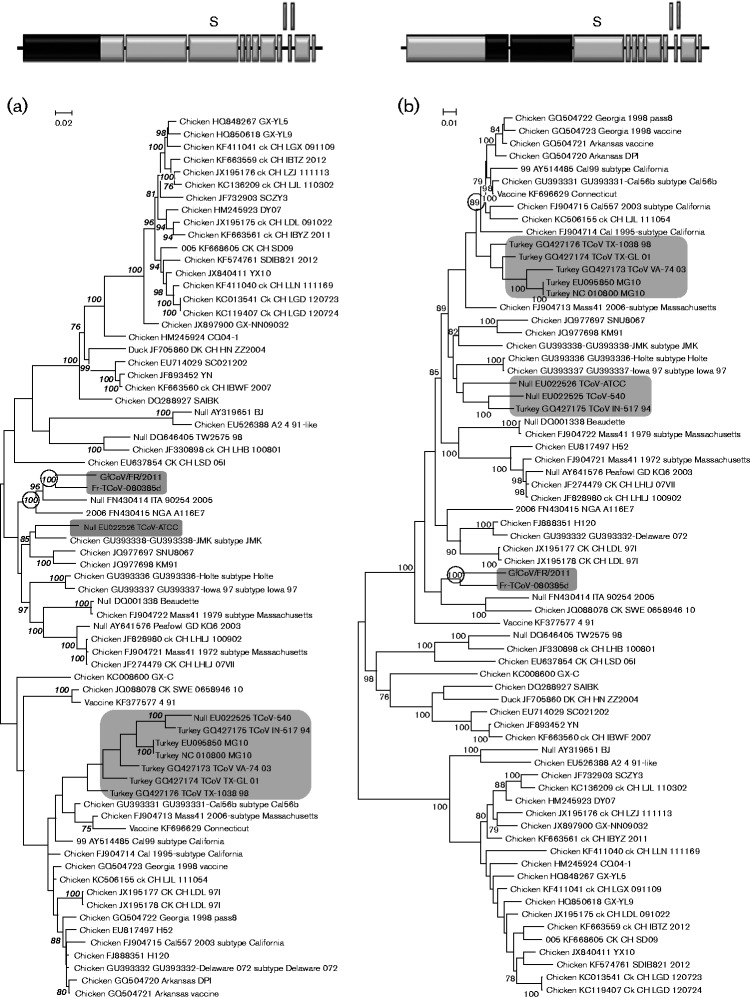

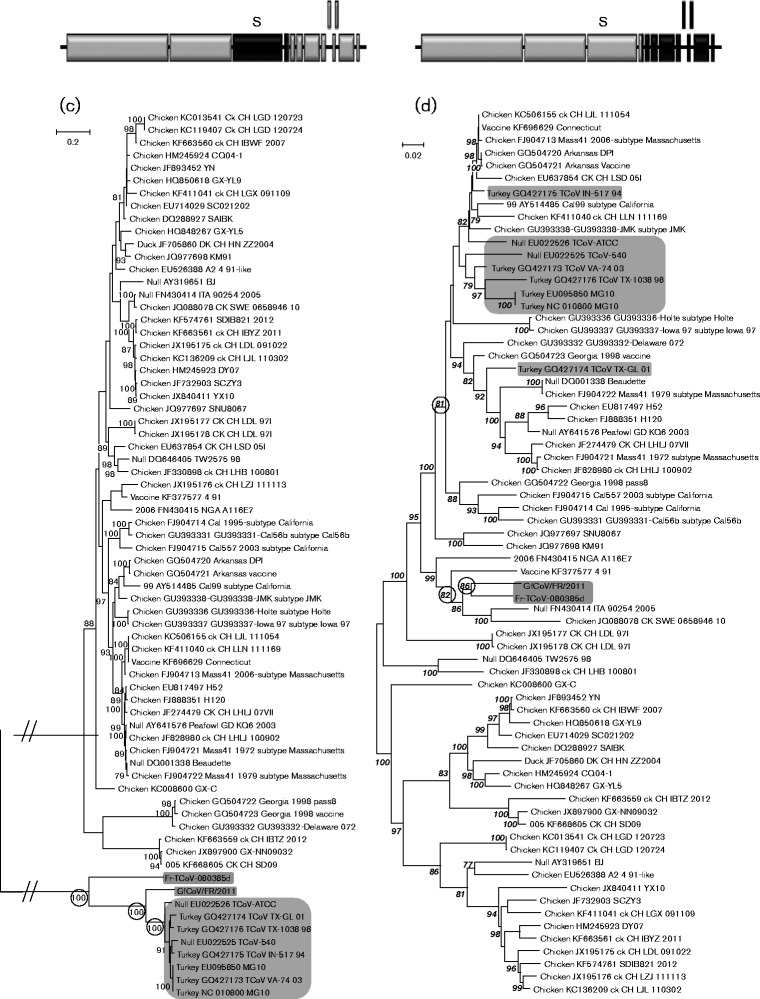
Phylogenetic relationships of the Fr TCoV genomic sequence assessed in four separate phylogenetic trees. Diagrams of the genome are provided above each tree and contain black shading to depict the segment of sequence used for that tree.

The full-length sequence of Fr TCoV was analysed phylogenetically in four segments based on the three predicted positions of recombination (Fig. 1). Each segment was aligned with corresponding zones taken from the 69 other full-length AvCoV sequences, to create four separate datasets. One tree was generated from each dataset (Fig. 2a–d).

In the tree generated using sequences 5′ to the recombination site predicted at position 8658 (Fig. 2a), Fr TCoV was clustered with European IBV (Italy 2005) and African IBV (NGA 2006) with a bootstrap value (bv) of 100 %, then subclustered with GfCoV/Fr/2011 (bv 100 %). Fr TCoV was not closely related with any of the American TCoVs. In the second tree (Fig. 2b), generated using nt 8659 to 20 280 (region between the first and second predicted recombinant sites), Fr TCoV was again coupled with GfCoV/Fr/2011 and again placed in close proximity to Italy 2005, although the bv (69 %) was below the threshold limit, set at 75 %. US TCoVs were grouped with IBVs of US origin (bv 85 %), with the exception of IBV Delaware 072. A unique trend was observed in the third tree (Fig. 2c), nt 20 281 to 24 058 (region between the second and third predicted recombinant sites, mostly corresponding to the S gene), where Fr TCoV, GfCoV/Fr/2011 and US TCoVs were grouped together (bv 100 %) and separated from all IBVs. These three types of viruses then formed three branches, each supported by a bv of 100 %. In the fourth tree (Fig. 2d), nt 24 059 to 27 739 (3′ part of the genome, downstream of the third predicted recombination site), Fr TCoV and GfCoV/Fr/2011 were again coupled together (bv 86 %), placed in a group containing European IBV, Italy 2005 (bv 82 %) and separated from the US TCoVs. US TCoVs were again grouped with IBVs of US origin (bv 100 %). In agreement with Fig. 2(c), plotting the single nucleotide polymorphisms between Fr TCoV, GfCoV/Fr/2011 and US TCoV or their closest IBV relative (Italy 2005) clearly showed that most genetic relatedness between Fr TCoV, GfCoV and US TCoV was linked to the S gene (Fig. S1, available in the online Supplementary Material).

**Fig. 3.**
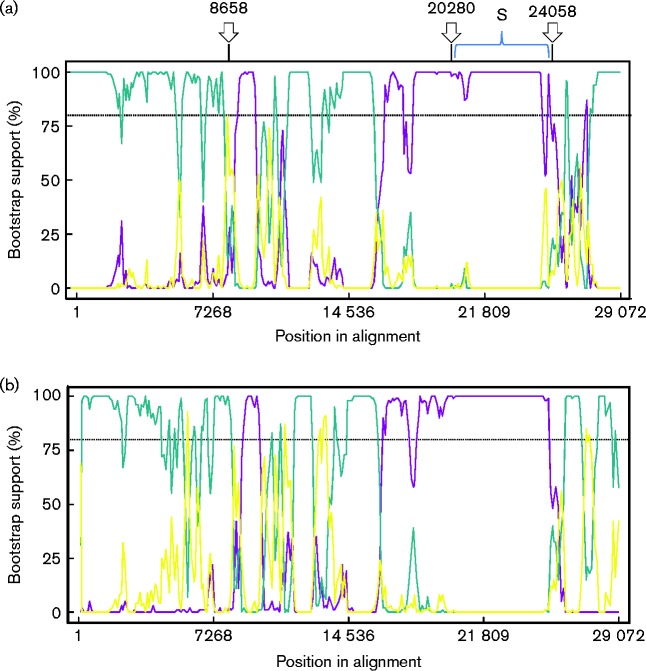
Bootscan analysis of the full-length genomic sequences of Fr TCoV, GfCoV/Fr/2011, US TCoV (TX-GL) and IBV (Italy 2005). In SimPlots (a) and (b) the query sequences are Fr TcoV and GfCoV respectively. Arrows above SimPlot (a) indicate putative breakpoints, which were predicted from analysis using the 69 full-length sequences. In (a) the yellow line corresponds to FN430414_ITA_90254_2005 – Turkey_GQ427174_TCoV_TX-GL_01; blue, FN430414_ITA_90254_2005 – Fr-TCoV-080385d_; purple, Turkey_GQ427174_TCoV_TX – GL_01 -Fr-TCoV-080385d_. In (b) the yellow line corresponds to FN430414_ITA_90254_2005 – Turkey_GQ427174_TCoV_TX-GL_01; blue, FN430414_ITA_90254_2005 – GfCoV/FR/2011_; purple, Turkey_GQ427174_TCoV_TX – GL_01 -GfCoV/FR/2011_. The dotted lines show bootstrap cut-offs of 80 %. Very similar plots were observed for FrTCoV and GfCoV in that both viruses were grouped with Italy 2005 for the majority of the sequences leading up to the S gene and after the S gene to the end of the genome.

To further illustrate the predicted recombination sites, a Bootscan analysis (Fig. 3) was performed using viruses that were closely related to Fr TCoV and GfCoV/Fr/2011 in the previously described phylogenetic trees. This analysis exposed traces that also grouped them with the European IBV Italy 2005 in the majority of the sequence leading up to the second predicted breakpoint at 20 280 (just upstream of the sequence encoding the S gene), and from the third predicted breakpoint, 24 058 (ORF 3a), to the end of the genome. A small zone of sequence (8747–9971) positioned around the first predicted breakpoint at 8658 (ORF 1a) grouped both Fr TCoV and GfCoV/Fr/2011 with US TCoV TX-GL. Again the zone of sequence encoding the S gene was the only zone grouping Fr TCoV and GfCoV/Fr/2011 with a US TCoV (US TCoV TX-GL).

### Time to the most recent common ancestor (TMRCA) analyses

**Fig. 4.**
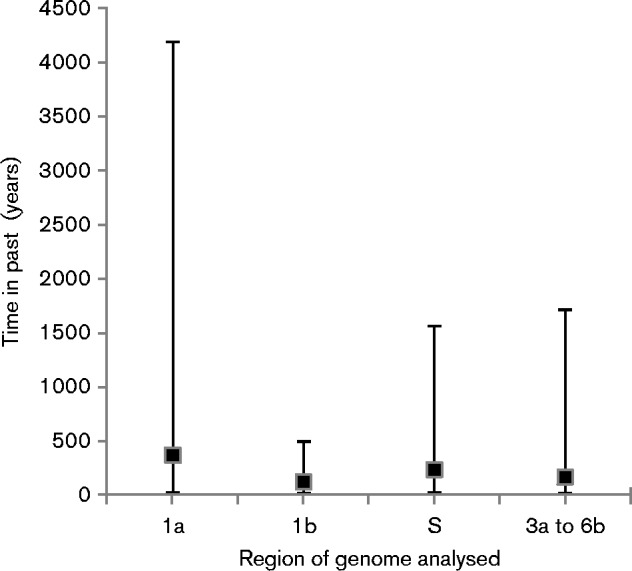
TMRCA analysis for different regions of the Fr TCoV and Gf CoV/Fr/2011 genomes. Black squares represent median values, and bars 95 % HPD intervals. Zero on the *y*-axis indicates the year when the most recent virus was isolated.

Fig. 4 summarizes the results of the TMRCA analyses. The mean TMRCA values for the 1a, 1b, S and 3a-to-6b regions were 363 [highest posterior density (HPD), 23–4192], 118 (HPD, 15–490), 232 (HPD, 19–1556) and 164 (HPD, 12–1710) years, respectively. All 95 % HPD confidence intervals were overlapping.

## Discussion

The aim of the present study was to determine the complete genome sequence of the only known TCoV isolated in Europe, so that further molecular characterization of the virus could be undertaken, to possibly document its evolution history compared with those of TCoVs isolated in the USA.

The overall length and organization of the Fr TCoV genome and the sizes of its putative ORFs 1a, 1ab, S, 3a, 3b, E, M, 4b, 4c, 5a, 5b, N and 6b agreed with those of US TCoVs and GfCoV/Fr/2011 published previously (Ducatez *et al.*, 2015; Gomaa *et al.*, 2008; Jackwood *et al.*, 2010), as did predicted protease cleavage sites in pp1ab. An investigation into the TRSs for these ORFs of Fr TCoV (as predicted from the genome sequence) revealed that they were identical to those of the US isolate TCoV-ATCC and GfCoV/Fr/2011, with the exception of the TRS proposed for ORF 6b (Table 1). It has been suggested that ORFs E and 6b use TRSs upstream of 3a and N respectively (Cao *et al.*, 2008); however, in the present study, specific TRSs are proposed for each ORF in light of the recent study that showed biological function of a non-canonical TRS in which only the last three bases (CAA) are required (Bentley *et al.*, 2013). In addition, the two TRSs for ORFs E and 6b only differed from the consensus sequence CUUAACAA in the first three bases (Table 1).

Previously, phylogenetic analysis based on either the S1 gene, a small portion of the N gene, or the RNA-dependent RNA polymerase gene from TCoV-positive field samples in France suggested different evolutionary pathways for TCoVs in Europe and North America, which, in agreement with a publication focusing on US TCoV (Hughes, 2011; Jackwood *et al.*, 2010), most likely came into being through recombination events (Maurel *et al.*, 2011). In the present study, investigations into the potential recombination events involved were undertaken, employing a recombination software package, RDP4 v4.39, in which different recombination detection programs are used. Three positions in the full-length Fr TCoV sequence were predicted with extremely robust *E*-values amongst six different detection methods, including Bootscan analysis, shown in Fig. 3. Moreover, the two sites flanking the S gene (20 280 and 24 058) were in line with the zones published previously for US TCoVs (20 173 and 23 849) (Jackwood *et al.*, 2010) and with recombination hotspots frequently found in IBV (Jackwood *et al.*, 2012).

Sequences between the three predicted positions of recombination were selected for individual phylogenetic analysis, producing the four trees shown in Fig. 2. In the tree produced with sequences 5′ to the putative recombination site predicted at position 8658 (Fig. 2a), Fr TCoV was closely related to GfCoV/Fr/2011 and another European coronavirus, IBV Italy 2005, all of which were genetically distinct from the US TCoVs, which were grouped with the US IBVs. This same pattern was observed in the trees in Fig. 2(b, d), which correspond to nt 8659 to 20 280 and 24 059 to 2 7739, respectively. The same trend, however, was not observed in the tree in Fig. 2(c) (nt 20 281 to 24 058). This region of sequence (mostly the S gene) resulted in one group containing Fr TCoV, GfCoV/Fr/2011 and US TCoVs that was separated from the IBV viruses. This TCoV/GfCoV/Fr/2011 cluster was further separated into Fr TCoV, GfCoV/Fr/2011 and US TCoVs. A calculation of nucleotide identities for this region between the three groups revealed a much closer relationship between GfCoV/Fr/2011 and US CoVs (80–81 %) than between GfCoV/Fr/2011 and Fr TCoV (64 %) or Fr TCoV and the US TCoVs (64–65 %). The Bootscan results mostly corroborated these relationships; however, they further identified a small zone of sequence (8747–9971) that grouped both Fr TCoV and GfCoV/Fr/2011 with an American TCoV, TCoV (TX-GL). This zone of sequence was not identified when using the 69 full-length sequences for prediction of recombination events; however, it did fall close to breakpoint 8658. As the two zones of sequence either side of this position grouped the same viruses, the first potential recombination breakpoint in terms of evolutionary hypothesis was considered to be position 20 281.

These phylogenetic data and identities are compatible with recombination events involving IBVs on different continents with several unknown CoVs. On the one hand, the S genes of GfCoV/Fr/2011 and US TCoV, and to a lesser extent Fr TCoV, share significant genetic relationships, and therefore these viruses must have acquired their S gene from a common genetic origin (unknown ancestor). On the other hand, GfCoV/Fr/2011 and Fr TCoV have a very similar genetic background in other genes, and therefore the recombination event that generated these viruses by conferring on them their specific S gene must have involved either the same or very similar recipient IBVs. However, although these new data do strengthen the case for recombination in the evolution of TCoVs and GfCoV, other explanations do remain possible until a source for the TCoV/GfCoV spike gene has been identified.

**Fig. 5.**
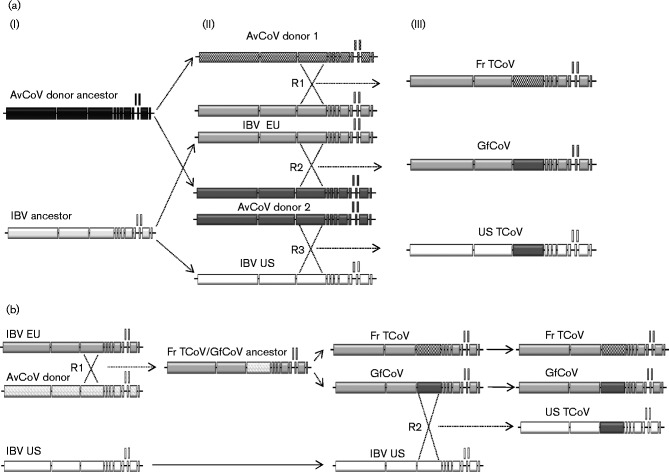
Hypothetical evolution of Fr TCoV, GfCoV/Fr/2011 and US TCoV. (a) From left to right: (i) IBV and AvCoV donor ancestors would have evolved to produce IBV EU and US plus AvCoV donors 1 and 2 respectively. (ii, iii) Three recombination events R1, R2 and R3 (iii) would have resulted in viruses Fr TCoV, GfCoV/Fr/2011 and US TCoV (iii). (b) From left to right: a recombination event (R1) between IBV EU and an AvCoV donor would have produced Fr TCoV/GfCoV/Fr/2011 ancestor, which would have evolved into FrTCoV and GfCoV/Fr/2011. Here, a recombination event (R2) between GfCoV/Fr/2011 and IBV US would have generated US TCoV.

In the evolutionary pathway proposed in Fig. 5(a), different recipient IBVs in Europe and the US gained a very similar S gene, from a common AvCoV donor (AvCoV donor 2), resulting in GfCoV/Fr/2011 and US TCoV, respectively [Fig. 5b (iii) lower panel)]. Independently from these events, the same European recipient IBV (or GfCoV/Fr/2011 itself) was involved in another recombination event with AvCoV donor 1, which supplied the Fr TCoV-like S gene, thus resulting in Fr TCoV [Fig. 5a (iii) upper panel]. A recombination event is proposed here rather than the evolution of GfCoV/Fr/2011 into Fr TCoV owing to the substantial number of nucleotide modifications required for GfCoV/Fr/2011 S to become Fr TCoV S. Also, as mutation rates in the S genes of IBV and TCoVs are very similar to those in their other genes (Hughes, 2011), then it would be expected that this number of changes would have imposed an accumulation of nucleotide modifications in the other areas of sequence, so that GfCoV and Fr CoV would not have been so closely related in the trees derived from other genes. Because of the similarities of the S genes of Fr TCoV, US TCoV and GfCoV/Fr/2011, it can be inferred that ‘AvCoV donor 2′ and ‘AvCoV donor 1’ should in this model share a common ancestor [‘AvCoV donor ancestor’ in Fig. 5a (i) upper panel]. Of course, all IBV strains participating in these recombination events do share a common ancestor [‘IBV ancestor’ in Fig. 5a (i) lower panel].

In the alternative evolutionary pathway proposed in Fig. 5(b), which could be considered simpler as it involves only two recombination events, a first recombination event (Fig. 5b, R1) occurred between an IBV EU recipient strain and an unknown AvCoV donor, resulting in a virus with a new S gene, whose evolution would have resulted in Fr TCoV and GfCoV/Fr/2011. A second recombination event (Fig. 5b, R2) involving a US IBV recipient and GfCoV/Fr/2011 would have generated US TCoV viruses, which share a stronger S gene similarity with GfCoV/Fr/2011 than with Fr TCoV.

To help evaluate which of the two hypotheses was the more plausible, attempts were made based on the sequence data presented here to define the TMRCA for the different regions of the studied genomes. A theoretical result showing a TMRCA for the S gene of Fr TCoV and GfCoV that was greater than the TMRCA of their proposed shared IBV backbones would mean that the hypothesis shown in Fig. 5(a) was more likely; however, a result showing a TMRCA for the S gene and the backbone that were the same would be in favour of Fig. 5(b). The actual results (Fig. 4), though, did not allow a clear conclusion to be drawn and therefore at this stage both evolutionary pathways are plausible.

Finally, both evolutionary pathways require that turkeys (a ‘New World’ bird) and guinea fowl (an ‘Old World’ bird) were raised in the same geographical location at some stage, or that they both came into contact with another species harbouring an ancestor common to GfCoV/Fr/2011 and TCoV. The domestication and complex worldwide diffusion of domestic guinea fowl, turkey and chickens may have provided ample opportunities over centuries for such inter-specific contacts. Chicken and guinea fowl breeding was already documented in ancient Greece, and guinea fowl meat featured in Roman banquets in the first century AD (Scherf, 2000). Although guinea fowl breeding was subsequently abandoned in Europe, this species was reintroduced when the Portuguese returned from Africa in the late 15th century (Belshaw, 1985). Both chicken and guinea fowl were transferred to the New World shortly after its Discovery: animal breeding farms established by 1495 in Cuba and Santo Domingo included chickens, which supposedly were shipped soon after to Mexico and South America (Scherf, 2000). The first shipments of guinea fowl to the Caribbean are documented slightly later, in 1508 (Lamblard, 1975). Such introductions of European poultry could have brought chicken and guinea fowl into contact with domestic turkeys, which apparently underwent a complex domestication history (Speller *et al.*, 2010; Kennedy Thornton *et al.*, 2012) and were reportedly widespread in Mesoamerica at the time of the Discovery and in the Caribbean soon after (Crawford, 1985). Reciprocally, the first shipment of domestic turkeys to Europe was documented in 1500 and turkey breeding, first encouraged in Spain in 1511, quickly expanded from southern to northern European countries in the first half of the 16th century (Crawford, 1985). Clearly, these movements must have led to the repeated introduction of naive birds into new ecosystems in both the New and Old Worlds, such epidemiological situations being favourable for inter-species transmission events. Similar situations most likely reoccurred over the centuries, as poultry (turkey) breeds developed in Europe were further re-exported to the Americas.

## Methods

### Viruses

Coronavirus Fr TCoV 080385d (Fr TCoV) used in this study was isolated from a field sample of digestive contents (duodenum) collected in France in November 2008 from 42-day-old turkeys. The virus was propagated and prepared as previously described, in embryonated eggs from specific-pathogen-free turkeys (Guionie *et al.*, 2013).

### RNA preparation for NGS

The Fr TCoV viral suspension was clarified with a 0.45 μm pore-size filter. Of this material, 340 μl was mixed with 10 μl DNase I (Qiagen) in buffer RDD (Qiagen) and 10 μl RNase Cocktail Enzyme Mix (Life Technologies), and incubated at 37 °C for 2 h to degrade all non-encapsidated nucleic acids. Then, RNA was extracted using TRIzol LS (Life Technologies) according to the manufacturer's instructions. This viral RNA sample was again treated with DNase (Turbo DNA-free; Life Technologies), followed by purification (RNeasy MinElute Cleanup kit; Qiagen). The size distribution of the extracted RNA fragments was checked by capillary electrophoresis using an Agilent 2100 Bioanalyzer and LabChip RNA 6000 Nano kit. cDNA synthesis and amplification was achieved using Ovation RNA-Seq System V2 (NuGEN). cDNA (1 μg) was sonicated in order to obtain fragments ranging from 150 to 500 bp. Finally, the library was constructed using the Encore Rapid DR Multiplex System 1–8 (NuGEN). NGS was done at the Biogenouest (Nantes, France) core facilities by using a MiSeq HD Sequencer (Illumina).

### Bioinformatic reconstruction of full-length genome

Sample reads were cleaned with Trimmomatic software (Bolger *et al.*, 2014).

First, a Bowtie2 (Langmead & Salzberg, 2012) alignment was compared with viral sequences in the ViPR database (Pickett *et al.*, 2012) to determine the closest viral genomes. Matching coronavirus sequences were downloaded to perform a second Bowtie2 alignment, which, when the coverage depth of the sequence was less than or equal to 30 replicates, was complemented by translated reads that matched existing reference IBV protein sequences. Five contigs were obtained in a draft assembly, which were manually fused into three contigs with the aid of blast. These three contigs had respective lengths of 13359, 541 and 13761 nt. A Bowtie2 alignment of cleaned reads was performed on these contigs to improve the consensus. Two small gaps in the sequence and the genome extremities remained undetermined at this stage.

### RNA preparation for RT-PCR and Sanger sequencing

Fr TCoV virus suspension (140 μl) (see above) was used to extract RNA on micro-centrifuge columns using a QIAmp viral RNA mini kit (Qiagen) according to the manufacturer's recommendations.

### Sanger sequencing: filling gaps and determination of the 5′ and 3′ genome extremities

All primers used in the following procedures are available on request from the authors.

Endpoint RT-PCR and Sanger sequencing were employed to complete two short regions (each approximately 20 nt) of sequence in ORF 1b that remained undetermined by NGS. First, cDNA molecules corresponding to the regions of missing sequence were prepared using Superscript II (Invitrogen) according to the manufacturer's recommendations. Next, dsDNA PCR products were amplified from each cDNA using Expand High Fidelity enzyme (Roche) according to the manufacturer's recommendations. Finally PCR products were purified using a NucleoSpin Gel and PCR Clean-up kit (Macherey Nagel) and sequenced using a BigDye Terminator v3.1 Cycle Sequencing kit as recommended by the manufacturer, in a 3130 automated sequencer (Applied Biosystems). Each PCR product was amplified twice and sequenced in both directions. The genome 3′ extremity was determined using classic 3′ RACE as previously described (Sambrook & Russell, 2001). Several attempts to determine the 5′ extremity using 5′ RACE failed; therefore, a consensus sequence derived from the 5′ extremities of 68 other AvCoVs was added.

### Phylogenetic analysis

Sixty-eight full-length genome sequences of AvCoVs (accession numbers provided in Table S1) were downloaded from the ViPR database (Pickett *et al.*, 2012), together with the full-length sequence of guinea fowl coronavirus (GfCoV/Fr/2011, accession no. LN610099) (Ducatez *et al.*, 2015), which was kindly provided by Dr M. Ducatez (INRA, Toulouse, France). The French TCoV sequence was aligned with these 69 sequences with clustal w. Alignment was adjusted and manually checked in accordance with the correct reading frame of the different genes. To optimize the phylogenetic analyses, the best-fitting nucleotide substitution model was evaluated using ‘Find Best DNA/Protein Models (ML)’ in mega v6 (Tamura *et al.*, 2013) according to the Akaike information criterion and Bayesian information criterion (Posada & Crandall, 2001). Then, phylogenetic analysis was performed by the maximum-likelihood method (Felsenstein, 1981) and the general time-reversible (GTR)+G+I substitution model. Bootstrap analysis (Felsenstein, 1985) of the resultant tree was performed using 1000 replicates. Only clusters with bv greater than or equal to 75 % were considered.

### Detecting recombination events

Recombination Detection software package RDP4 v4.39 (Martin *et al.*, 2010), in which different recombination detection programs (rdp, geneconv, SiScan, Bootscan, MaxChi, Chimaera and 3Seq) are included, was used to detect potential recombination events between all available full genomes of avian gammacoronaviruses (*N* = 69). Recombination events were considered reliable when detected with the highest multiple-comparison corrected *P*-value cut-off set at 10^− 5^ in at least four different methods. In order to expose the recombination events implicating Fr TCoV and GfCoV/Fr/2011 by Bootscan analysis, three US TCoVs (MG10, TX-GL 01 and VA-74 03), three IBVs closely related to Fr TCoV (Italy 2005, NGA A116E7 and SWE 065894) and three more distant IBV relatives (Beaudette, KM 91 and 4/91) were used. The analysis was performed on the basis of pairwise distance, modelled with a window size of 1000 nt, step size of 100 nt, and 100 bootstrap replicates as parameters (Fig. 3).

Sequences between the predicted recombination breakpoints of Fr TCoV were assessed in four phylogenetic trees as described above. One tree corresponded to nt 0–8658 (region 5′ to the recombination site predicted at position 8658; Fig. 1), the second to nt 8659 to 20 280 (region between the first and second predicted recombinant sites), the third to nt 20 281 to 24 058 (region between the second and third predicted recombinant sites) and the fourth to 24 059 to 27 739 (3′ part of the genome, downstream of the third predicted recombination site). These four trees are shown in Fig. 2.

### TMRCA analyses

The TMRCA of avian gammacoronaviruses was estimated based on four different genome regions (part of 1a, part of 1b, the full-length S gene and part of the region after S). For each partial genome region, all dated sequences that were neither of clone nor of vaccine origin and did not expose any evidence of recombination were selected (typically 40 sequences per region). For the S gene, all available full-length sequences of TCoV and GfCoV/Fr/2011 that were dated were used, corresponding to seven American TCoV, four French TCoV and GfCoV/Fr/2011. With BEAUti software, the uncorrelated log-normal relaxed molecular clock with the GTR+G + I model of nucleotide substitution was applied with constant size demographic models. The trace files were visualized with Tracer 1.6, especially to verify that the effective sample size value was greater than 200, which corresponds to the minimum acceptable number of independent samples. Maximum clade credibility trees were generated after removing a 10 % burn-in with TreeAnnotator v1.7.5. The trees obtained were also visualized and annotated by FigTree v1.4 software. For each part of the genome studied, the age of the most recent common ancestor between Fr TCoV and GfCoV/Fr/2011 was determined with a posterior probability above or equal to 0.90, and then dates for the different genome regions were compared. Uncertainty in the estimated results was reported as values of the 95 % HPD.

### Comparing nucleotide sequences

ORFs were predicted for Fr TCoV using the Vigor software (Wang *et al.*, 2010) and Vector NTI Advance 11 (Invitrogen) to analyse the composition of its genes and their lengths. Protease cleavage recognition sites in the polyprotein 1ab were predicted using ZCurve software (http://tubic.tju.edu.cn/sars/) (Gao *et al.*, 2003).

## Supplementary Data

306 Supplementary Data
