# Supplementary material for: First complete genome sequence of European turkey coronavirus suggests complex recombination history related with US turkey and guinea fowl coronaviruses
Source: J Gen Virol. 2016 Jan;97(Pt 1):110–20. doi: 10.1099/jgv.0.000338 (PMC7081074; doi:10.1099/jgv.0.000338)
Supplement: Supplementary file 1 — Supplementary Data [file jgv-97-110-s001.pdf]

Table S1 : Accession N° and names of sequences used in this study

Accession No	ID Name
AY514485	IBVCal99 Subtype California 99
AY319651	IBV BJ
AY641576	IBVPeafowl GD KQ6 2003
DQ001338	IBVBeaudette
DQ288927	IBVSAIBK Chicken
DQ646405	IBVTW2575 98
EU022525	Turkey coronavirus TCoV-540
EU022526	Turkey coronavirus TCoV-ATCC
EU095850	Turkey coronavirus MG10 Turkey
KR822424	Fr-TCoV-080385d
EU526388	IBVA2 4 91-like Subtype 4 91-like Chicken
EU637854	IBVCK CH LSD 05I Chicken
EU714029	IBVSC021202 Chicken
EU817497	IBVH52 Chicken
FJ888351	IBVH120 Chicken
FJ904713	IBVMass41 2006-Massachusetts Chicken
FJ904714	IBVCal 1995-California Chicken
FJ904715	IBVCal557 2003-California Chicken
FJ904721	IBVMass41 1972-Massachusetts Chicken
FJ904722	IBVMass41 1979-Massachusetts Chicken
FN430414	IBVITA 90254 2005
FN430415	IBVNGA A116E7 2006
GQ427173	Turkey coronavirus TCoV VA-74 03 Turkey
GQ427174	Turkey coronavirus TCoV TX-GL 01 Turkey
GQ427175	Turkey coronavirus TCoV IN-517 94 Turkey
GQ427176	Turkey coronavirus TCoV TX-1038 98 Turkey
GQ504720	IBVArkansas DPI Chicken
GQ504721	IBVArkansas Vaccine Chicken
GQ504722	IBVGeorgia 1998 pass8 Chicken
GQ504723	IBVGeorgia 1998 Vaccine Chicken
GQ504725	IBVMass41 Vaccine Chicken
GU393331	IBV-GU393331-Cal56b Subtype Cal56b Chicken
GU393332	IBV-GU393332- Delaware 072 Chicken
GU393336	IBV-GU393336-Holte Chicken
GU393337	IBV-GU393337-Iowa 97 Chicken
GU393338	IBV GU393338-JMK Chicken
HM245923	IBVDY07 Chicken
HM245924	IBVCQ04-1 Chicken
HQ848267	IBVGX-YL5 Chicken
HQ850618	IBVGX-YL9 Chicken
JF274479	IBVCK CH LHLJ 07VII Chicken
JF330898	IBVck CH LHB 100801 Chicken
JF705860	IBVDK CH HN ZZ2004 Duck
JF732903	IBVSCZY3 Chicken
JF828980	IBVck CH LHLJ 100902 Chicken
JF893452	IBVYN Chicken
JQ088078	IBVCK SWE 0658946 10 Chicken
JQ977697	IBVSNU8067 Chicken
JQ977698	IBVKM91 Chicken
JX195175	IBVck CH LDL 091022 Chicken
JX195176	IBVck CH LZJ 111113 Chicken
JX195177	IBVCK CH LDL 97I Chicken
JX195178	IBVCK CH LDL 97I Chicken
JX840411	IBVYX10 Chicken
JX897900	IBVGX-NN09032 Chicken
KC008600	IBVGX-C Chicken
KC013541	IBVCk CH LGD 120723 Chicken
KC119407	IBVCk CH LGD 120724 Chicken
KC136209	IBVck CH LJL 110302 Chicken
KC506155	IBVck CH LJL 111054 Chicken
KF377577	IBV4 91 vaccine
KF411040	IBVck CH LLN 111169 Chicken
KF411041	IBVck CH LGX 091109 Chicken
KF574761	IBVSDIB821 2012 Chicken
KF663559	IBVck CH IBTZ 2012 Chicken
KF663560	IBVck CH IBWF 2007 Chicken
KF663561	IBVck CH IBYZ 2011 Chicken
KF668605	IBVCK CH SD09 005
KF696629	IBVConnecticut vaccine
NC010800	IBVMG10 Turkey

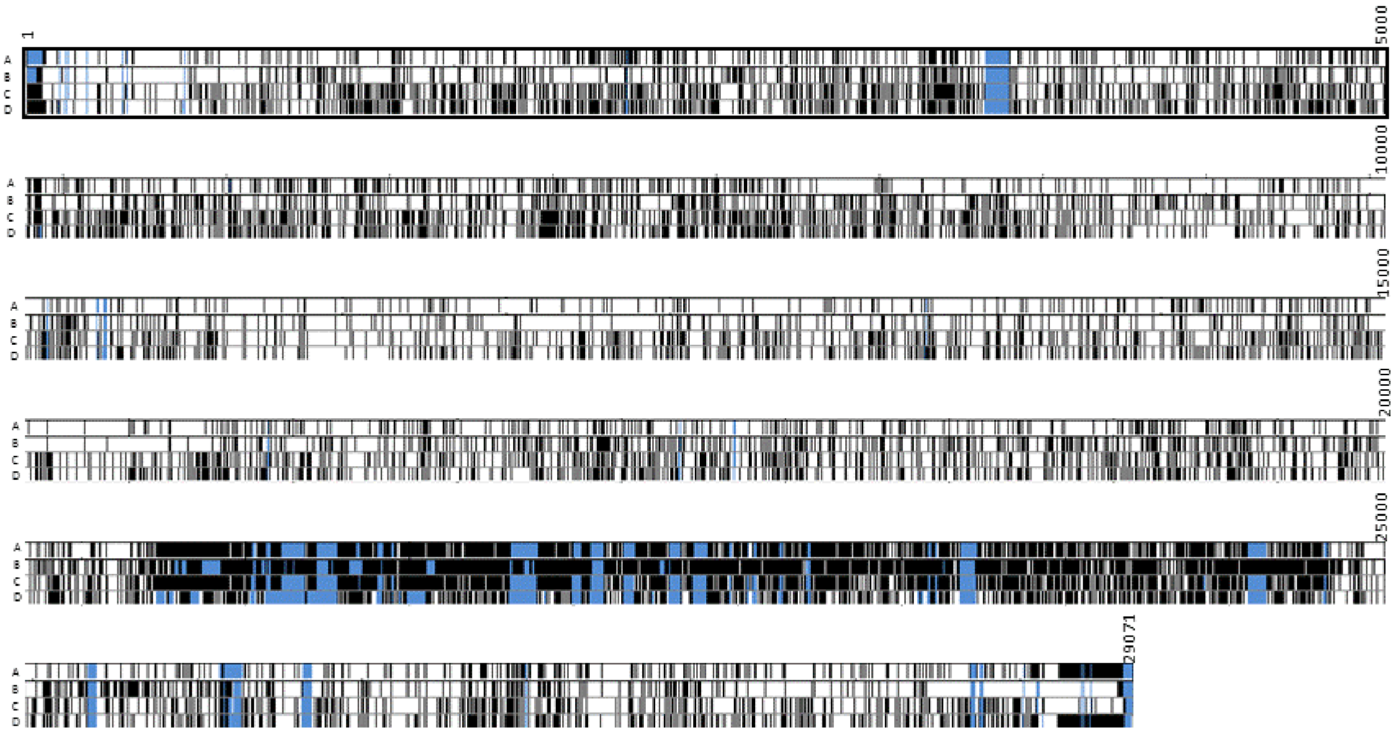

Suppl Fig 1. : Repartition of mutations between FrTCoV/GfCoV/ITA05/American TCoV based on an alignment of 69 full length avian gammacoronavirus genomes. A) Mutations observed between FrTcoV and GfCoV. B) Mutations observed between FrTCoV and ITA05. C) Mutations observed between FrTCoV and a consensus sequence of American TCoVs. D) Mutations observed between GfCoV and a consensus sequence of American TCoV. For each comparison, black regions correspond to the SNPs and blue regions correspond to a presence of gaps in two compared sequences
